# Health Effects of Soy Isoflavones and Green Tea Catechins on Cancer and Cardiovascular Diseases Based on Urinary Biomarker Levels

**DOI:** 10.3390/molecules27248899

**Published:** 2022-12-14

**Authors:** Tomokazu Ohishi, Noriyuki Miyoshi, Mari Mori, Miki Sagara, Yukio Yamori

**Affiliations:** 1 Institute of Microbial Chemistry (BIKAKEN), Numazu, Microbial Chemistry Research Foundation, Shizuoka 410-0301, Japan; 2 Laboratory of Oncology, Institute of Microbial Chemistry (BIKAKEN), Microbial Chemistry Research Foundation, Tokyo 141-0021, Japan; 3 Graduate School of Integrated Pharmaceutical and Nutritional Sciences, University of Shizuoka, Shizuoka 422-8526, Japan; 4 Department of Health Management, School of Health Studies, Tokai University, Kanagawa 259-1292, Japan; 5 NPO World Health Frontier Institute, Nishinomiya 663-8143, Japan; 6 Institute for World Health Development, Mukogawa Women’s University, Nishinomiya 663-8143, Japan; 7 Disease Model Cooperative Research Association, Kyoto 606-0805, Japan

**Keywords:** soy isoflavones, genistein, green tea catechins, EGCG, urinary biomarkers, cancer, cardiovascular diseases

## Abstract

Plant polyphenols have various health effects. Genistein, which is abundant in soybeans, and epigallocatechin-3-gallate, which is abundant in green tea, are major flavonoids, a subclass group of polyphenols. Several epidemiological studies have shown that these flavonoids have beneficial effects against cancer and cardiovascular diseases. However, other studies did not show such effects. Several confounding factors, including recall bias, are related to these inconsistent findings, and the determination of metabolites in the urine may be useful in reducing the number of confounding factors. Equipment, which can be used by research participants to collect samples from a portion of voided urine within 24 h without the help of medical workers, has been developed for epidemiological investigations. Previous studies, in which flavonoid metabolites in these urine samples were measured, revealed that soy intake was correlated with a reduced risk of certain types of cancer and cardiovascular diseases worldwide. Although soybeans and green tea consumption may have protective effects against cancer and cardiovascular diseases, further clinical studies that consider different confounding factors are required to provide evidence for the actual impact of dietary flavonoids on human diseases, including cancer and cardiovascular diseases. One possible mechanism involved is discussed in relation to the downregulation of reactive oxygen species and the upregulation of 5′-adenosine monophosphate-activated protein kinase elicited by these flavonoids.

## 1. Introduction

**Figure 1:**
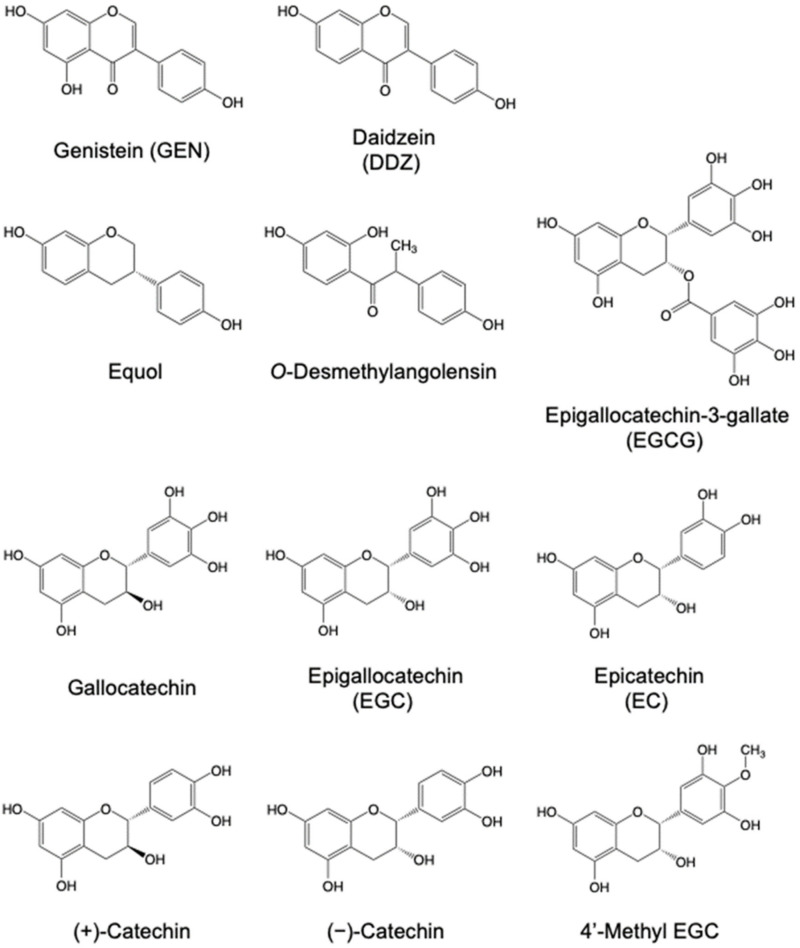
Chemical structures of representative flavonoids and their urinary metabolites.

Plant polyphenols are found in foods including vegetables, fruits, legumes, nuts, and beverages. Genistein (GEN), which is abundant in soybeans, and epigallocatechin-3-gallate (EGCG), which is abundant in green tea, are major flavonoids, a subclass group of polyphenols (Figure 1) [1]. In a cohort study conducted by Wang et al. [2], the median intake of total flavonoids ranged from 8.88 mg/day in the lowest quintile to 47.44 mg/day in the highest quintile among 38,408 middle-aged and older women.

Several studies have shown a correlation between flavonoid-rich diets and a reduced risk of cancer, cardiovascular and neurodegenerative diseases, and other conditions [1,3]. For example, a meta-analysis conducted by Woo and Kim [4] evaluated 35 observational epidemiological studies. Results showed that the intakes of total dietary flavonoids and most subclasses were inversely associated with a risk of smoking-related cancers including bladder, esophageal, laryngeal, liver, lung, oral, pancreatic, renal, and stomach cancers.

Tsugane et al. [5] emphasized that ischemic heart disease (IHD) and cancer have low mortality rates in populations with a low intake of red meat, particularly saturated fatty acids. Furthermore, high intakes of fish, plant foods such as soybeans, and non-sugar-sweetened beverages including green tea may contribute to the longest average life expectancy in Japan, which is among the G7 countries.

However, several epidemiological studies did not show such beneficial effects on health. The inconsistent results may have been caused by recall bias and incomplete or inadequate adjustments of confounding factors, such as tobacco smoking, the use of different ingredients, cooking/brewing temperature, and genetic characteristics [6,7,8,9]. Evaluating the contribution of diet to disease risks requires accurate assessments of dietary exposure based on nutritional epidemiologic studies [10]. One problem is that the assessment methods used to measure the quantity of compound intakes significantly varies [11].

The measurements of flavonoids and/or their metabolites in the blood and urine can provide more accurate data on the consumption of flavonoids. We searched PubMed, Web of Science, and Google Scholar to collect human and cell-based studies about cancer and cardiovascular diseases (CVDs) from 1983 to 2022, and about 70% of the retrieved studies were from the last 10 years. We included studies with both positive and negative results on the preventive potential of soy isoflavones and green tea catechins against cancer and CVDs by focusing on human studies to examine the urinary metabolites of isoflavones and catechins to identify the beneficial effects of soy food and green tea consumption on these diseases.

## 2. Epidemiological Studies on the Anticancer Effects of Flavonoid-Rich Foods

### 2.1. Epidemiological Studies of the Anticancer Effect of Soy Consumption

Several epidemiologic studies have found that a soy-rich diet reduces the risk of cancer, and isoflavones such as GEN and daidzein (DDZ) can have anticancer effects [8,12,13]. In vitro experiments have shown that these compounds have anticancer effects, and they can be used as therapeutic agents for the treatment of certain cancer types [14,15].

A dose–response meta-analysis of prospective cohort studies, which included 23 studies with a sample size of 330,826 participants, revealed that soy/soy product consumption was inversely associated with cancer-related mortality (pooled relative risk (RR) = 0.88; 95% confidence interval (CI) = 0.79–0.99) [16]. The risk of gastric, colorectal, and lung-cancer-related mortality decreased, and an increase in the intake of soy isoflavones by 10 mg/day was associated with a 7% and 9% decreased risk of mortality from all cancers and breast cancer (BCa), respectively.

Grosso et al. [17] conducted meta-analyses and their results showed that the intake of isoflavones was significantly associated with a decreased risk of lung and stomach cancers and nearly significantly correlated with breast and colorectal cancers based on prospective studies. Furthermore, it has been associated with ovarian, breast, colorectal, endometrial, and lung cancers according to case–control studies. Based on a meta-analyses of 97 cohort studies on the association between dietary intake and the risk of ovarian cancer, the consumption of flavonoids was associated with a significantly low risk of this cancer (RR = 0.83, CI = 0.78–0.89) [18].

Wang et al. [19] performed a population-based case–control study from 2010 to 2011 in Connecticut. The results showed that women who consumed GEN at a dose of 1860–3110 μg/day had a lower risk of papillary macrocarcinomas than women who consumed GEN at a dose of <760 μg/day (odds ratio (OR) = 0.26, 95% CI = 0.08–0.85). A meta-analysis conducted by Applegate et al. [20] revealed that the pooled RR of prostate cancer (PCa) was 0.90 (95% CI = 0.84–0.97) in patients with GEN consumption.

A population-based case–control study examined the association between dietary flavonoid consumption and the risk of esophageal squamous cell carcinoma (ESCC) among adults from a high-risk area in China. The results showed that when comparing the highest and lowest intake quartiles, an inverse association was observed between the risk of ESCC and the consumption of isoflavones (OR = 0.34; 95% CI = 0.23–0.50), DDZ (OR = 0.31; 95% CI = 0.21–0.45), and GEN (OR = 0.34; 95% CI = 0.23–0.50) [21].

A case–control study on 249 incident PCa cases and 404 matched controls found an inverse association for DDZ urinary excretion (OR for the highest vs. lowest quintile = 0.55, 95% CI = 0.31–0.98). Moreover, a weaker inverse association was found for GEN urinary excretion [22].

In a prospective nested case–control study in Japan, an inverse association was noted between plasma GEN levels, but not DDZ, and the risk of BCa. However, nonsignificant inverse associations were found between the risk of BCa and GEN and DDZ consumption [23].

A meta-analysis of two randomized controlled trials (RCTs) found a significant reduction in the diagnostic rate of PCa after the consumption of soy/soy isoflavones (risk ratio = 0.49; 95% CI = 0.26–0.95) [24]. A meta-analysis conducted by Yan and Spitznagel [25] showed that the intake of isoflavones was not associated significantly with the risk of PCa. However, separate analyses showed that the combined RRs/ORs were 0.52 (95% CI = 0.34–0.81) in studies on Asian populations and 0.99 (95% CI = 0.85–1.16) in studies on Western populations, thereby indicating ethnic differences.

A meta-analysis of eight studies found that compared with the lowest level of soy food intake (≤5 mg isoflavones per day), the risk of BCa was reduced weakly among individuals with a modest intake of isoflavones (ca. 10 mg per day) (OR = 0.88; 95% CI = 0.78–0.98) and strongly among those with a high intake of isoflavones (≥20 mg per day) (OR = 0.71; CI = 0.60–0.85) [26]. However, no association was found in studies on 11 Western populations with low soy consumption (average highest and lowest soy isoflavone intake levels of 0.8 and 0.15 mg/day, respectively).

A systematic review of 11 epidemiological studies showed that two cohort and two case–control studies indicated a moderate to strong and a weak inverse association, respectively, between soy consumption and the risk of BCa in postmenopausal Japanese women [27]. Similarly, Messina et al. [28] showed a significantly low risk in the development of BCa with soy consumption during childhood and/or adolescence.

Ponte et al. [29] and the National Clinical Trial Number summarized the clinical trials based on the administration of flavonoids including GEN, DDZ, and catechins for cancer. The results provided sufficient evidence supporting flavonoids as important adjuvants in cancer therapy and more comprehensive information on the anticancer effects of the flavonoids.

### 2.2. Evaluation of the Anticancer Effects of Soy Consumption Based on Urinary Biomarker Levels

#### 2.2.1. Urinary Biomarker Levels According to Soy Consumption

Urinary isoflavonoid excretion is associated with soy consumption. The intake of soy foods was correlated with urinary concentrations of GEN (*r* = 0.40; *p* = 0.0001), O-desmethylangolensin (O-DMA) (*r* = 0.37; *p* = 0.0002), and DDZ (*r* = 034; *p* = 0.0007), and the sum of isoflavones (*r* = 0.39; *p* = 0.0001) [30]. Equol is a metabolite generated by the gut microbiome from DDZ, and its excretion is limited to 20–30% of Western populations and 50–70% of Asian populations [31]. A cross-sectional study conducted by Maskarinec et al. [32] showed a strong correlation between urinary isoflavone excretion and self-reported soy intake. DDZ is metabolized to equol and O-DMA by intestinal bacteria in approximately 30–50% and 80–90% of individuals, respectively [33].

Based on data from the US Department of Agriculture isoflavone database and dietary recalls of 2908 American adults with urinary isoflavone data in the 1999–2002 National Health and Nutrition Examination Survey, Chun et al. [34] found that the mean isoflavone intake was 1.0 mg/day. The isoflavones taken were GEN (55%), DDZ (35%), glycitein (7%), biochanin A (2%), and formononetin (2%). The mean urinary isoflavone concentration was 5.0 ng/mL among isoflavone consumers, and the urinary GEN and DDZ excretion was correlated with isoflavone intake levels.

The urinary excretion rate of total isoflavonoids was significantly correlated with dietary soy food intake (a correlation coefficient of approximately 0.5). Hence, the urinary excretion rate of total isoflavonoids in overnight urine samples may reflect the usual intake of soy foods [35].

#### 2.2.2. Cancer Epidemiology Based on Urinary Isoflavone Metabolites

In a case–control study, women with newly diagnosed early-stage BCa and controls were interviewed using questionnaires, and 72 h urine and blood samples were collected [36]. In 144 pairs, a high equol excretion was significantly associated with a low risk of BCa, and the ORs according to quartiles were 1.00, 0.45 (95% CI = 0.20–1.02), 0.52 (95% CI = 0.23–1.17), and 0.27 (95% CI = 0.10–0.69). Increased urinary DDZ concentrations were associated with an insignificant reduction in the risk of BCa. In this study, the GEN assay was not carried out. Hence, the usefulness of urinary GEN is not known.

In a study of 117 case–control pairs of postmenopausal women in Shanghai, Dai et al. [37] found a reduced risk of BCa among women with a high urinary isoflavonoid excretion rate.

The urinary excretion rates of isoflavonoids, mammalian lignans, and citrus flavonoids were analyzed using overnight urine samples collected from 250 patients with incident BCa and matched controls. The results showed that patients with BCa had significantly lower urinary excretion of total isoflavonoids than controls [38]. The risk of BCa was reduced with increased excretion of total isoflavones with adjusted ORs of 0.62 (95% CI = 0.39–0.99) for the highest versus the lowest tertile of the total excretion.

**Figure 2:**
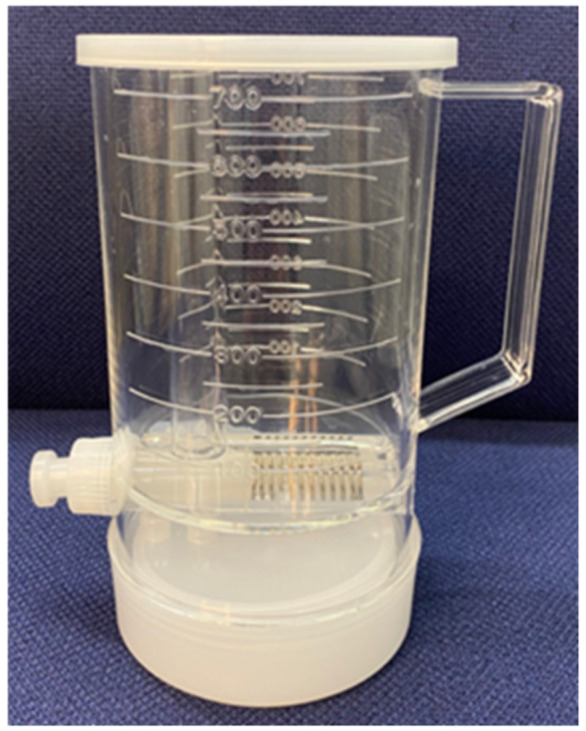
An aliquot cup that allows one-40th of urine samples to be collected personally at each time of voiding during 24-h urine sampling [39,43].

**Figure 3:**
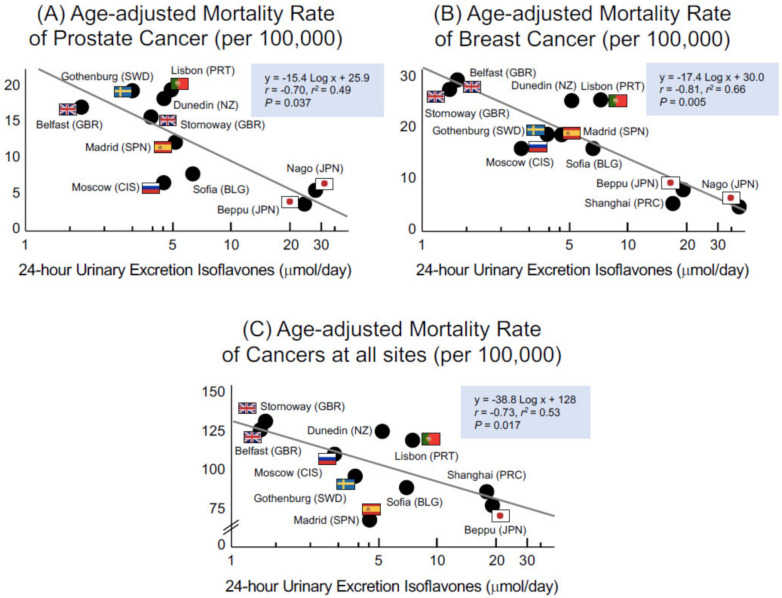
Relationship between 24 h urinary isoflavone excretion and age-adjusted mortality rates of PCa (**A**), BCa (**B**), and cancers at all sites (**C**) [41,42]. Each vertical axis represents age-adjusted mortality rate per 100,000 persons.

Yamori et al. [39,40] developed a piece of equipment called an “aliquot cup” for collecting urine samples. Hence, research participants can accurately collect their samples from a portion of 24 h urine without the help of a physician for epidemiological studies (Figure 2). This cup can collect a 2.5% aliquot of urine into the lower compartment after urine is voided into the upper compartment within 24 h. With this device, the lower rate of mortality from PCa, BCa, and all cancers in Okinawans was shown to be inversely correlated with 24 h isoflavone excretions in the WHO–CARDIAC study populations (Figure 3) [41,42]. This worldwide survey revealed that soy food dishes rich in isoflavones could be useful in preventing cancer.

Analyses of urine samples collected from 251 women with BCa and 462 controls revealed that the risk of BCa was inversely associated with increased urinary excretion of total isoflavones (OR = 0.80 for the 25th–75th percentile; 95% CI = 0.65–0.99) and GEN (OR = 0.88 for the 25th–75th percentile; 95% CI = 0.78–0.99) [44]. In Japanese-American women, a significant reduction in the risk of BCa was associated with the highest quartile of urinary DDZ (OR = 0.41; 95% CI = 0.19–0.89). No significant association was found between the risk of BCa and equol excretion.

A clinical study aimed to examine equol-producing status in relation to breast density in 224 women aged 36–58 years. The results showed that 30% were equol producers who had a significantly lower mean percentage of breast density than nonproducers (32% vs. 35%, *p* = 0.03) [45]. The dietary effects of isoflavones on BCa may be dependent on the ability to metabolize DDZ to equol.

A prospective cohort study on 88 BCa cases and 268 controls in 14,697 postmenopausal women showed that a higher urinary GEN excretion was insignificantly associated with a reduced risk of BCa [46].

Li et al. [47] performed a prospective cohort study of 478 women with incident lung cancer and matched controls. Results showed that a moderate intake of dietary isoflavones was inversely associated with lung cancer risk in never-smoking women. Further, analyses of urinary biomarkers showed that the ORs for the second, third, and fourth quartiles were 0.57 (95% CI = 0.39–0.83), 0.64 (95% CI = 0.44–0.92), and 0.60 (95% CI = 0.41–0.86).

A clinical study found that higher levels of urinary GEN and DDZ were associated with a reduced risk of advanced-stage endometriosis but not early endometriosis [48]. For advanced-stage endometriosis, the adjusted ORs of the highest quartile group were 0.21 (95% CI = 0.06–0.76) for GEN and 0.29 (0.08–1.03) for DDZ. Hence, the intake of soy foods can have preventive effects against ovarian cancer in view of the malignant potential of endometriosis [49].

A nested case–control study conducted in Shanghai, China, showed that the adjusted ORs of liver cancer across increasing quartiles of urinary GEN levels were 1.00 (reference), 0.55 (95% CI = 0.22–1.36), 0.57 (95% CI = 0.23–1.43), and 0.19 (95% CI = 0.06–0.59) in women and 1.00 (reference), 1.22 (95% CI = 0.52–2.86), 1.17 (95% CI = 0.47–2.90), and 1.23 (95% CI = 0.55–2.76) in men [50]. These findings indicate that the urinary excretion of GEN may be associated with a reduced risk of liver cancer in women but not in men.

In a hospital-based case–control study in Jamaica that analyzed urine samples, men who produced equol had a decreased risk of total PCa (for tertile 2: OR = 0.42; 95% CI = 0.23–0.75) (for tertile 3: OR = 0.48; 95% CI = 0.26–0.87) and high-grade disease (for tertile 2: OR = 0.31; 95% CI = 0.15–0.61) (for tertile 3: OR = 0.29; 95% CI = 0.13–0.60) than those who are nonproducers (reference tertile) [51]. No association was found between the urinary excretion of GEN and DDZ and the risk of PCa.

Song et al. [52] examined the association between DDZ-metabolizing phenotypes and mammographic density. The results showed that the mammographic density was 39% lower in equol producers than in nonproducers. The producers of O-DMA derived from DDZ had a >69% greater mammographic density than nonproducers. Therefore, the intestinal bacterial profiles are associated with postmenopausal mammographic density, which can be correlated with the risk of BCa.

A cross-sectional study evaluated participants aged 48–82 years. The results showed that equol producers (30% of participants) with weekly soy intake had a lower % mammographic density, an intermediate marker of BCa risk. Meanwhile, in nonproducers, weekly soy intake was associated with a higher % density [53]. Therefore, dietary soy had different effects on breast tissue between equol producers and nonproducers.

In contrast, Seow et al. [54] found no associations between the frequency of overall soy intake and the levels of urinary equol and O-DMA in spot samples. Thus, these biomarkers in a spot sample may not reflect current soy consumption in epidemiological studies.

In a clinical study that evaluated serum and urine samples, Grace et al. [55] found that the levels of serum DDZ, serum equol, and urinary equol were significantly associated with an increased risk of BCa, although the intake of GEN and DDZ was correlated with a reduced risk of PCa. There was no significant correlation between urinary equol and the risk of PCa [22].

Larger clinical studies are necessary to determine the potential anticancer effects of isoflavones and the detection method associated with the prevention of cancer.

### 2.3. Epidemiological Studies on the Anticancer Effects of Green Tea

Several epidemiological studies revealed that green tea consumption has beneficial effects against cancers [6,9,15]. Recent studies have supported these effects as exemplified by the following findings:

Zhao et al. [56] performed a meta-analysis of prospective cohort studies. The results showed an inverse association between green tea intake and lymphoid neoplasm (RR = 0.72; 95% CI = 0.52–0.98) if three studies were combined. A higher green tea intake was associated with a decreased risk of glioma (RR = 0.81; 95% CI = 0.70–0.95) if six studies were combined. A lower risk of bladder cancer was detected for every one cup increase in the intake of tea (RR = 0.95; 95% CI = 0.91–0.99) when five studies were combined, and for gastric and esophagus cancers, the RRs of two cohorts were combined (RR = 0.85; 95% CI = 0.73–0.99). Similarly, Pranata et al. [57] found an association between higher tea consumption and a lower risk of glioma (RR = 0.84; 95% CI = 0.71–0.98). The risk of glioma decreased by 3% with one cup of green tea per day.

A pooled analysis based on the findings in 9438 cases and 20,451 controls from 22 studies worldwide found that the adjusted pooled OR of regular tea drinkers was 0.91 (95% CI = 0.85–0.97) [58]. Stronger inverse associations were detected among regular drinkers who consume green tea frequently in China and Japan (OR = 0.67; 95% CI = 0.49–0.91).

A dose–response analysis of the association between green tea consumption and esophageal cancer was performed. The results showed no association between esophageal cancer and one cup increase in the consumption of green tea per day (OR 1.00; 95% CI = 95–1.04)**.** In the subgroup analysis, the OR was 0.79 in women (95% CI = 0.68–0.91). Therefore, green tea has a protective effect in women [59].

A comprehensive review by Abe and Inoue [60] showed that most meta-analyses found inverse associations between green tea consumption and endometrial cancer (RR = 0.89; 95% CI = 0.84–0.94) [61], lung cancer (OR = 0.69; 95% CI = 0.48–0.82) [62], non-Hodgkin’s lymphoma (RR = 0.61; 95% CI = 0.38–0.99) [63], oral cancer (RR = 0.85; 95% CI = 0.78–0.93) [64], and ovarian cancer (RR = 0.64; 95% CI = 0.45–0.90) [65]. Meanwhile, mixed results were observed in breast, esophageal, gastric, and hepatic cancers. However, a null association was noted in colorectal, pancreatic, and prostate cancers.

Based on a meta-analyses of 97 cohort studies on the association between dietary intake and the risk of ovarian cancer, the consumption of green tea was significantly associated with a reduced cancer risk (RR = 0.61; 95% CI = 0.49–0.76) [18].

However, several previous and recent studies did not show the anticancer effect of tea consumption. For example, in Asian populations, green tea consumption was not associated with lower risks of cancer-related mortality, although an association was found in mortality from all causes and coronary heart disease (CHD) [66]. According to a randomized, double-blind clinical trial on groups taking decaffeinated green tea catechin extract (GTE) with 150 mg of EGCG or placebo for 3 years, GTE was correlated with a slightly lower adenoma rate. However, the result was not statistically significant [67].

### 2.4. Evaluation of the Anticancer Effects of Green Tea Consumption Based on Urinary Biomarker Levels

#### 2.4.1. Urinary Metabolites in Tea Catechins

Human studies on the absorption, distribution, metabolism, and excretion of tea catechins found that urinary excretion of metabolites during a 24 h period after green tea consumption corresponded to 28.5% of the ingested (epi)catechin (EC) and 11.4% of (epi)gallocatechin (EGC). Thus, it has a higher absorption than other flavonoids [68].

An intervention study evaluated participants who consumed green tea polyphenol (GTP) capsules daily at doses of 500 and 1000 mg or a placebo for 3 months, and 24 h urine samples were examined. The results showed that the urinary excretions of EGC and EC can be practical biomarkers of green tea consumption in human studies [69].

A clinical investigation evaluated the correlation between the dietary intake of polyphenol-rich foods and metabolites in 24 h urine samples collected from 419 participants. The results showed that urinary concentrations of (+)-catechin and EC are useful when used as short-term nutritional biomarkers of dietary catechin, EC, and total flavan-3-ol monomers [70].

An intervention study evaluated healthy postmenopausal women who are at high risk of BCa and who received GTE corresponding to 843.0 ± 44.0 mg EGCG/day or a placebo for 1 year. The results showed that the urinary levels of catechins including EGC and EC were similar between the GTE and placebo groups at baseline. However, after treatment, the GTE group had significantly higher urinary catechin levels than the placebo group [71]. The GTE group had a 10.6-fold increase in urinary EGC levels and 16.5-fold increase in EC.

#### 2.4.2. Cancer Epidemiology Based on Urinary Metabolites of Tea Catechins

Yuan et al. [72] found that the ORs of colon cancer in the lowest, intermediate, and highest tertiles of urinary EGC were 0.64 (95% CI = 0.33–1.24), 0.60 (95% CI = 0.30–1.20), and 0.40 (95% CI = 0.19–0.83), respectively. For urinary 4′-methyl EGC, the ORs of colon cancer in the second, third, and fourth quartiles were 0.49 (95% CI = 0.25–0.96), 0.32 (95% CI = 0.16–0.67), and 0.41 (95% CI = 0.20–0.84), respectively. No association was observed between the urinary levels of EC or its metabolite and the risk of colon cancer. The risk of rectal cancer was not associated with urinary biomarker levels.

A nested case–control study aimed to investigate the association between urinary tea catechin markers and the risk of gastric and esophageal cancers on 190 incident cases of gastric cancer and 42 cases of esophageal cancer. The results showed that urinary EGC was significantly inversely associated with gastric cancer (OR = 0.52; 95% CI = 0.28–0.97) after adjusting for confounders including *Helicobacter pylori* infection [73]. Similar associations were observed if gastric cancer and esophageal cancer sites were combined. Therefore, tea catechins may act as chemopreventive agents against gastric and esophageal cancers.

Green tea may have a greater protective effect against the risk of BCa among women with the low-activity-associated genotype of the catechol-*O*-methyltransferase (COMT), which may modulate the urinary excretion of tea catechins [74]. In 660 cohort participants, men with the homozygous low-activity-associated COMT genotype had lower urinary levels of the metabolite of tea polyphenols and all five tea polyphenol metabolites than those with the high-activity-associated COMT genotypes [74]. Therefore, men with the low-activity-associated COMT genotype can excrete fewer tea polyphenols, thereby indicating that they may retain more tea polyphenols in their bodies and gain greater health benefits from green tea intake.

Based on a study of 353 patients with BCa and 701 matched controls in a cohort of women aged 40–70 years at baseline, the urinary excretion of EC was inversely associated with the risk of BCa (OR = 0.59; 95% CI = 0.39–0.88 for the intermediate tertile) [75].

In the Shanghai Women’s Health Study on BCa, Luo et al. [76] found an inverse association between urinary EGC levels and the risk of BCa among those null for glutathione S-transferase M1 (GSTM1), particularly for both *GSTM1* and *GSTT1*.

In contrast, a nested case–control study of hepatocellular carcinoma (HCC) (211 cases and 1067 matched controls) in the Shanghai Cohort Study found a statistically significant association between high urinary EGC levels and a high risk of HCC among participants who tested positive for the hepatitis B surface antigen [77]. High levels of catechins may be associated with an increased risk of HCC in high-risk individuals.

## 3. Preventive Effects of Isoflavones against CVDs

### 3.1. Epidemiological Studies on the Preventive Effects of Soy Consumption against CVD

Shirota et al. [78] recently performed a systematic review on Japanese-style diet with the highly ranked components of soy foods, green tea, seafood, vegetables, rice, miso soup, seaweed, pickles, and fruits. The results showed that the risk of mortality decreased in individuals with CVD, cerebrovascular disease (stroke), and IHD according to the characteristic Japanese food intake.

A prospective cohort study of approximately 75,000 Chinese women found a dose–response relationship between soy food intake and the risk of CHD with an adjusted RR of 0.25 (95% CI = 0.10–0.63) in the highest versus the lowest quartile of total soy protein intake [79].

A meta-analysis of cohort studies showed that soy/soy product consumption was inversely associated with CVD-related mortality (pooled effect size = 0.85; 95% CI = 0.72–0.99) [16]. However, the intake of soy protein was not significantly associated with mortality from all causes and CVD.

In a cohort study with 4,826,122 person years of follow-up, isoflavone intake was inversely associated with CHD (pooled hazard ratio (HR) = 0.87; 95% CI = 0.81–0.94) compared with the extreme quintiles [80]. The consumption of tofu, a soy product, had an evident effect on lowering the risk of CHD in young and postmenopausal women who do not use hormones.

In a review article, based on a series of evidence from observational studies and RCTs, Sekikawa et al. [31] revealed that soy isoflavones, particularly equol, are antiatherogenic, and they can improve arterial stiffness and may prevent CHD.

In a cohort study with 503,998 person years of follow-up, Kokubo et al. [81] found that the multivariate HRs of soy intake of ≥5 times per week versus 0–2 times per week were 0.64 (95% CI = 0.43–0.95) for cerebral infarction, 0.55 (95% CI = 0.26–1.09) for CHD, and 0.31 (95% CI = 0.13–0.74) for mortality from cerebral infarction. The HRs for the highest versus the lowest quintiles of isoflavones in women were 0.35 (95% CI = 0.21–0.59) for cerebral infarction, 0.37 (95% CI = 0.14–0.98) for myocardial infarction (MI), and 0.87 (95% CI = 0.29–2.52) for CVD mortality. Thus, a high isoflavone intake was associated with a reduced risk of CI and MI in Japanese women. However, no significant association was observed between the dietary intake of soy and isoflavones and CI and MI in men.

A prospective cohort study of approximately 75,000 Chinese women found a dose–response relationship between soy food intake and the risk of CHD, with an adjusted RR of 0.25 (95% CI = 0.10–0.63) in the highest versus the lowest quartile of total soy protein intake [79].

A case–control study on 191 patients with ischemic stroke (IS) in eastern China found a significant inverse association between fruit consumption (OR = 0.29; 95% CI = 0.18–0.46) and soy consumption (OR = 0.47; 95% CI = 0.29–0.75) and IS [82].

In a study of 890 Japanese men, a high total soy product intake within 12 weeks was associated with significantly lower levels of low-density lipoprotein cholesterol (LDL-C) [83].

In a meta-analysis of randomized double-blind placebo-controlled trials, Mosallanezhad et al. [84] found a significant improvement in systolic blood pressure (SBP) (weighted mean difference: −1.70 mmHg; 95% CI = −3.34 to −0.06) and diastolic blood pressure (DBP) (weighted mean difference: −1.27 mmHg; 95% CI = −2.36 to 0.19 mmHg) after soy consumption.

Based on a cross-sectional study on 10,536 children and adolescents aged 7–18 years in southern China, 39.5% of participants had a high soy food intake (more than three times per week), which was significantly associated with a lower prevalence of hypertension and a greater prevalence of obesity [85].

In contrast, several studies did not show an inverse association between soy isoflavone intake and CVDs. For example, Yan et al. [86] performed a meta-analysis of 10 prospective cohorts and 7 case–control studies with 17,269 CVD events. The results showed no associations between soy isoflavone consumption and the risk of CVD, stroke, and CHD. However, they found a significant inverse association between soy intake and the risk of CVD (summary RR = 0.84, 95% CI = 0.75–0.94). Similarly, a meta-analysis of five prospective cohorts and six case–control studies found a significant inverse association between soy intake and the risk of stroke (summary RR = 0.54; 95% CI = 0.34–0.87) and CHD (summary RR = 0.66; 95% CI = 0.56–0.77) in case–control studies but not in prospective cohort studies [87]. No association was noted between soy isoflavone intake and the risk of stroke and CHD. Thus, data on the association between soy isoflavone intake and the risk of CVDs are contrasting.

A cross-sectional study was performed on 648 postmenopausal women who provided DDZ-challenged urine samples for determining the production of equol and O-DMA, another metabolite of DDZ. The results showed that soy isoflavone intake had minimal correlation with cardiovascular risk factors in producer phenotypes of these metabolites [88]. Other studies did not show the beneficial effects of dietary soy intake on CVDs [89,90].

### 3.2. Preventive Effects of Soy Consumption Based on Urinary Biomarker Levels

**Figure 4:**
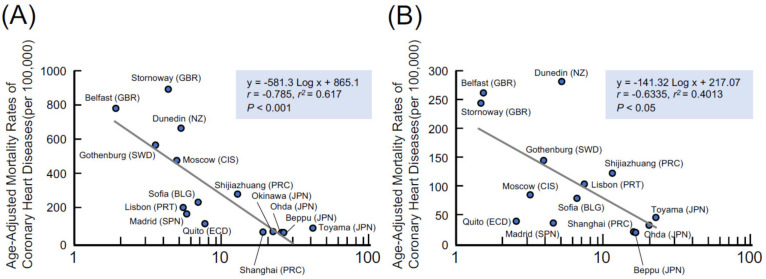
Relationship between 24 h urinary excretion of isoflavones and age-adjusted coronary heart disease mortality rates in men (**A**) and women (**B**). Modified data from a study [42] are presented.

In worldwide epidemiological surveys conducted on 61 populations in 25 countries, Yamori [42] showed that soybean diets contribute to healthy longevity via the prevention of CVDs in the Japanese population. This finding was correlated to 24 h urinary excretions of isoflavones [41,42]. Figure 4 presents the association between the age-adjusted CHD mortality rate and 24 h urinary isoflavones excretion, thereby indicating that the consumption of soy foods will be useful for preventing CHD worldwide.

Yamori et al. [91] performed a clinical study on 553 inhabitants aged 30–79 years in Hyogo Prefecture, Japan. The results showed that when quantified urinary isoflavones (mainly DDZ) excreted in the 24 h urine sample were separated into three tertiles and adjusted for age and sex, the highest tertile showed significantly higher levels of high-density lipoprotein cholesterol (HDL-C), total cholesterol, and 24 h urinary sodium and potassium than the lowest tertile. Therefore, a high consumption of soy may contribute to the low CHD mortality rate and high serum HDL-C levels in Japanese populations.

In a later study, the biomarkers of Japanese diets were analyzed in comparison with 24 h urine samples collected for grading Japanese diets by soy isoflavones and seafood taurine excretions [40]. A higher excretion of both biomarkers was associated with a lower risk of obesity, decreased serum cholesterol levels, and high HDL-C levels, thereby reducing the risk of CHD. However, it was associated with a higher salt intake, which causes hypertension, thereby leading to higher stroke risks.

A 2-year intervention study on 351 women showed that isoflavone supplementation reduced LDL-C levels by approximately 6% [92].

A 6-month RCT on 270 eligible female equol producers showed that the serum LDL-Cs of the whole soy and DDZ groups decreased by 7.95% (95% CI = −15.09–−0.81%) and 6.32% (95% CI = −13.45–0.08%), respectively. Furthermore, their serum C-reactive protein decreased by 0.164 (95% CI = −0.309–−0.019) and 0.054 (95% CI = −0.199–0.012), respectively [93].

In an intervention study in Scotland, 61 men aged 45–59 years with relatively high blood pressure (BP) and/or total cholesterol levels had diets containing at least 20 g of soy protein and 80 mg of isoflavones for 5 weeks [94]. The results showed a significant difference in 24 h urinary isoflavone excretion between the two groups and significant reductions from the baseline levels of BP, TC, and non-HDL-C levels in the soy group.

In accordance with this finding, a study covering 60 populations in 25 countries showed that soy intake was significantly associated with decreased body mass index, BP, and serum cholesterol levels. Furthermore, the CHD mortality rates were inversely correlated with isoflavones in 24 h urine samples [42,95].

By reviewing 69 articles, Zhang et al. [96] found that equol possesses antioxidative, antiinflammatory, and vasodilatory properties, and they may have a greater cardioprotective benefit than soy isoflavones themselves. Zhang et al. [97] conducted a case study. The results showed that the total urinary isoflavonoid levels were not associated with CHD, and urinary equol excretion was significantly inversely associated with CHD in women, with adjusted ORs for CHD, across increasing quartiles of the equol levels (0.61, 95% CI = 0.32–1.15; 0.51, 95% CI = 0.26–0.98; and 0.46, 95% CI = 0.24–0.89).

A prospective cohort study on 66,832 Chinese women found that a regular high intake of soy isoflavones may be associated with an increased risk of IS. However, there was no association between this risk and urinary isoflavone concentrations [98]. This difference may be attributed to the availability of single spot urine samples alone.

In a cross-sectional study, the participants received supplementation with one soy bar per day for 3 days. A urine analysis revealed that 17.5% of participants were equol producers [99]. The results showed no significant associations between equol production status and overall breast pathology, ductal hyperplasia, and BCa.

## 4. Preventive Effects of Green Tea against CVDs

### 4.1. Epidemiological Studies on Preventive Effects of Green Tea Consumption against CVDs

As previously mentioned, a recent meta-analysis of cohort studies revealed a mortality risk reduction in CVD, stroke, and/or heart disease with the intake of characteristic Japanese foods including green tea [78]. In another study, a linear meta-regression analysis of data from 39 prospective cohort studies on adults aged ≥18 years revealed that an increase in tea consumption (236.6 mL per day) was associated with a 4% lower risk of CVD-related mortality, 2% lower risk of CVD events, 4% lower risk of stroke, and 1.5% lower risk of all-cause mortality [100]. Hence, daily tea intake may be associated with lower risks of CVD and all-cause mortality among adults.

In meta-analyses of prospective observational studies, a dose–response analysis indicated that tea consumption had protective effects against stroke (RR = 0.96; 95% CI = 0.94–0.99), IS (RR = 0.76; 95% CI = 0.69–0.84), and hemorrhagic stroke (RR = 0.79; 95% CI = 0.72–0.87) [101]. In the Norfolk cohort of the European Prospective Investigation into Cancer, a minimal, but significant, inverse association was observed between the risk of CVD and the intake of EC in men (HR = 0.92; 95% CI = 0.86–0.99) [102].

A meta-analysis of 22 prospective studies including 8459 CHD cases, 10,572 stroke cases, 5798 cardiac death cases, 2350 stroke death cases, and 13,722 total death cases found that increased tea consumption by three cups per day was associated with a reduced risk of CHD (RR = 0.73; 95% CI = 0.53–0.99), cardiac-related mortality (RR = 0.74; 95% CI = 0.63–0.86), stroke (RR = 0.82; 95% CI = 0.73–0.92), total mortality rate (RR = 0.76; 95% CI = 0.63–0.91), cerebral infarction (RR = 0.84; 95% CI = 0.72–0.98), and intracerebral hemorrhage (RR = 0.79; 95% CI = 0.72–0.87) [103]. However, tea intake had no or minimal effects on stroke mortality.

Grosso et al. [104] showed that black tea and green tea supplementation was associated with a significant reduction in both SBP (−1.04 mmHg, 95% CI = −2.05 to −0.03 and −1.17 mmHg, 95% CI = −2.18 to −0.16, respectively) and DBP (−0.59 mmHg, 95% CI = −1.05 to −0.13 and −1.24 mmHg, 95% CI = −2.07 to −0.40), respectively, based on 13 clinical trials (*n* = 1115) and 24 trials (*n* = 1697).

A Japanese cohort study with an 18.5-year median follow-up showed that green tea consumption was inversely associated with all-cause mortality among CVD survivors. The multivariate HRs of stroke were 0.73 (95% CI = 0.42–1.27) for 1–6 cups/week, 0.65 (95% CI = 0.36–1.15) for 1–2 cups/day, 0.56 (95% CI = 0.34–0.92) for 3–4 cups/day, 0.52 (95% CI = 0.31–0.86) for 5–6 cups/day, and 0.38 (95% CI = 0.20–0.71) for ≥7 cups/day [105]. A similar inverse association was observed in CVD survivors, but not in those without a history of stroke or CVD.

Samavat et al. [106] conducted an RCT study in which participants were randomly assigned to receive GTE or a placebo and consumed either four GTE or placebo capsules daily for 12 months. The results revealed that compared with the placebo, supplementation with GTE significantly reduced the levels of circulating TC (−2.1% vs. −0.7%), LDL-C (−4.1% vs. 0.9%), and non-HDL-C (−3.1% vs. −0.4%). There was no change in HDL-C concentration. However, the triglyceride concentrations increased by 3.6% in the GTE group.

Xu et al. [107] performed a meta-analysis of five clinical trials on 408 individuals. The results showed that regular tea intake reduced SBP (−4.81 mmHg; 95% CI = −8.40 to −1.58) and DBP (−1.98 mmHg; 95% CI = −3.77 to −0.20). The analysis based on the categorization of tea type resulted in the finding that the hypotensive effects of green tea were more evident than those of black tea.

In a meta-analysis of 24 trials with 1697 participants, the pooled results revealed that green tea significantly lowered systolic BP (−1.17 mmHg; 95% CI = −2.18–−0.16 mm Hg) and diastolic BP (−1.24 mmHg; 95% CI = −2.07 to −0.40 mmHg). However, significant heterogeneity was found in both SBP and DBP [107].

A 5-year prospective cohort study of 3870 participants found that participants were less likely to be habitual tea drinkers if they were in the groups with an SBP of 144.4–149.9, 157.2–180.0, or 184.7–209.8 mmHg than those in the group with an SBP of 125.3–130.0 mm Hg. Thus, habitual tea consumption may give favorable effects on SBP [108].

A population-based cohort of 38,913 Chinese participants with a median follow-up of 5.9 years found that habitual tea drinkers (≥3 times/week for at least 6 months) had a 17% lower risk of high BP progression (OR = 0.83, 95% CI = 0.79–0.88) and a 14% decreased risk of incident hypertension (HR = 0.86, 95% CI = 0.80–0.91) compared with nonhabitual tea drinkers [109].

Yang et al. [110] conducted a cohort study on 1507 participants in Taiwan. The results showed that the risk of hypertension decreased by 46% among individuals who consumed 120–599 mL of tea per day at least for 1 year and by 65% among those who consumed 600 mL/day. A two-sample Mendelian randomization approach to 40,585 stroke cases and 406,111 controls revealed that an extra daily cup of tea was inversely associated with a risk of small vessel stroke (OR = 0.79; 95% CI = 0.69–0.91), but not with cardioembolic stroke, large artery stroke, stroke, and IS [111].

In contrast, previous studies have reported several contrasting results. For example, in two large cohort studies, Feng et al. [112] found that habitual tea drinking is associated with a slightly higher risk of hypertension and a minor increase in BP among middle-aged and older Chinese adults. Peng et al. [113] revealed that Chinese longevous men who drink green tea daily had a 38% higher risk of developing hypertension. Nevertheless, green tea had no effect in women.

### 4.2. Epidemiological Studies of Anti-CVD Effects of Tea Consumption Evaluated Using Urinary EGCG Metabolites

There are no epidemiological studies on the effect of tea consumption on CVDs based on urinary biomarker levels.

### 4.3. Mechanistic Aspects

**Figure 5:**
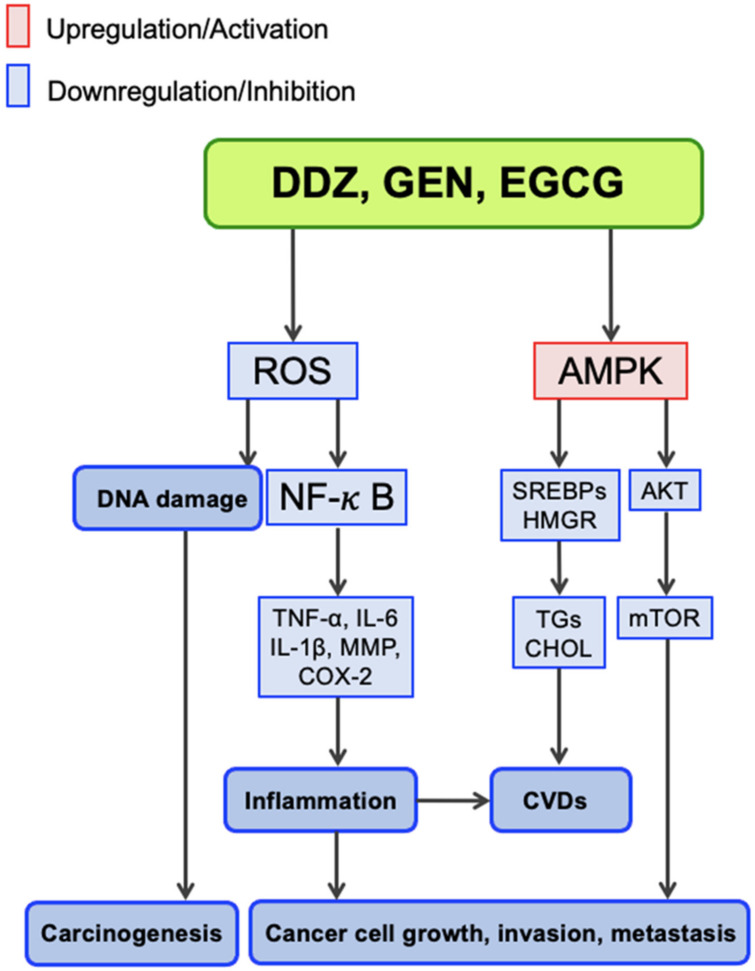
A putative major mechanism by which flavonoids exhibit anti-CVD and anticancer effects.

The current review focuses on epidemiological evidence for the effects of soy/isoflavones and green tea/EGCG on cancer and CVDs. Accordingly, we briefly discuss the molecular mechanisms associated with these effects. Ponte et al. [29] summarized the molecular mechanisms of flavonoids against cancer and the role of inflammation inhibition, modulation of NF-κB, MAPK, JAK-STAT, mammalian target of rapamycin (mTOR), and Ras signaling, apoptosis induction, and promotion of cell cycle arrest. We previously discussed the anticancer mechanisms of these polyphenols and underscored their antioxidative properties in the reactive oxygen species (ROS)-mediated signaling pathways (Figure 5) [6,15]. DDZ, GEN, and EGCG are strong antioxidants and can reduce oxidative stress by scavenging ROS, leading to the suppression of DNA damage [6,15,114,115]. Scavenging ROS also leads to the downregulation of the transcription factor NF-κB, resulting in the suppression of inflammatory cytokine gene expression, including TNF-α, IL-1β, and IL-6. Suppression of MMPs and COX-2 also elicits antiinflammatory effects that lead to anticancer effects. In addition, the dietary flavonoids activate 5′-adenosine monophosphate-activated protein kinase (AMPK). Activated AMPK can downregulate mTOR [116], leading to the suppression of tumorigenesis [117]. The antiinflammatory properties of these dietary flavonoids contribute to their anti-CVD mechanism, as described in several recent comprehensive reviews [114,115]. The anti-CVD mechanisms include the downregulation of NF-κB. The regulation of lipid metabolism is also a major concern in CVDs [114,115]. The activation of AMPK downregulates sterol response element-binding proteins (SREBPs), leading to the suppression of triglyceride and cholesterol synthesis and the stimulation of lipolysis [118,119].

Figure 5 depicts one of the most important mechanisms by which flavonoids elicit anti-CVD and anticancer effects, based on our data and previously published data [6,15].

## 5. Discussion

Several epidemiological studies have shown that isoflavone-rich diets have beneficial effects against several diseases such as cancer and CVDs.

Animal and cell-based studies have supported these findings. However, other human studies did not show that isoflavone has such health benefits. As previously mentioned, the contrasting results might be attributed to recall bias and from incomplete or inadequate adjustments of various confounding factors [6,7,9]. Variations in the assessment methods for quantifying the intakes of these compounds could have contributed to these results [11]. Therefore, the analysis of blood and urinary biomarkers may reduce recall bias, thereby leading to more comprehensive data on the effects of soy isoflavones and green tea catechins.

Several studies have shown some confounding factors. Yan and Spitznagel [25] revealed differences in the effects of isoflavones on the risk of PCa between Asian and Western populations, thereby indicating difference in the effects in terms of genetics. This finding may be correlated with the notion showing that equol excretion is limited to 20–30% of Western populations and 50–70% of Asian populations [31]. Inoue-Choi et al. [74] revealed that the bioavailability of green tea polyphenols may be dependent on the COMT genotype.

In a study conducted by Zhang et al. [50], an inverse association between urinary GEN excretion and the risk of liver cancer was detected in women but not in men, thereby indicating differences in terms of sex and hormonal status. Kokubo et al. [81] found an inverse association between isoflavone intake and cerebral infarction-, CHD-, and CVD-related mortality in women but not in men. The differences in sex hormones, physical characteristics, lifestyle behaviors, and dietary habits between men and women may contribute to some of the different results obtained from men and women. Tseng et al. [45] showed that the effects of isoflavones on BCa may be dependent on the ability to metabolize DDZ to equol. Therefore, comprehensive analyses are required in a larger research population to clarify the different data.

Studies on urinary biomarkers have contrasting results. Yu et al. [98] showed the limitation of single spot urine samples. Thus, 24 h urine samples can be used to obtain more reliable results. As shown in Figure 2 and Figure 3, a clear inverse association was observed between urinary isoflavones and cancer and CVDs based on different studies worldwide. Notably, urinary isoflavones can have an inverse association with salt excretion [91]. Nevertheless, future clinical studies accounting for these confounding factors could provide further evidence for the impacts of dietary flavonoids on human diseases, including cancer and CVDs.

## Data Availability

Not applicable.

## Abbreviations

AKT	Protein kinase B
AMPK	5′ adenosine monophosphate-activated protein kinase
BCa	Breast cancer
BP	Blood pressure
CHD	Coronary heart disease
CI	Confidence interval
COMT	Catechol-O-methyltransferase
COX-2	Cyclooxygenase-2
CVDs	Cardiovascular diseases
DBP	Diastolic blood pressure
DDZ	Daidzein
EC	Epicatechin
EGC	Epigallocatechin
EGCG	Epigallocatechin-3-gallate
ESCC	Esophageal squamous cell carcinoma
GEN	Genistein
GSTM1	Glutathione S-transferase M1
GTE	Green tea catechin extract
HCC	Hepatocellular carcinoma
HDL	High-density lipoprotein cholesterol
HDL-C	HDL-cholesterol
HMGR	3-Hydroxy-3-methylglutaryl-coenzyme A reductase
HR	Hazard ratio
IHD	Ischemic heart disease
IL-1β	Interleukin-1β
IL-6	Interleukin-6
IS	Ischemic stroke
JAK	Janus kinase
LDL	Low-density lipoprotein
LDL-C	LDL cholesterol
MAPK	Mitogen-activated protein kinase
MI	Myocardial infarction
MMP	Matrix metalloproteinase
mTOR	Mammalian/mechanistic target of rapamycin
NF-κB	Nuclear factor-κB
O-DMA	O-desmethylangolensin
OR	Odds ratio
PCa	Prostate cancer
RCTs	Randomized controlled trials
ROS	Reactive oxygen species
RR	Relative risk
SBP	Systolic blood pressure
SREBPs	Sterol regulatory element-binding proteins
STAT	Signal transducer and activator of transcription
TGs	Triglycerides
TNF-α	Tumor necrosis factor-α
WHO	World Health Organization
